# Enhancing effects of *Aloe vera* gel extracts on the humoral and cellular immune response and growth performance in broiler chickens

**DOI:** 10.5455/javar.2024.k745

**Published:** 2024-03-12

**Authors:** Mohammad Alam Miah, Snigda Akter, Md. Saif Uddin, Khaled Mahmud Sujan, Afrina Mustari, Sharmin Akter

**Affiliations:** Department of Physiology, Bangladesh Agricultural University, Mymensingh, Bangladesh

**Keywords:** *Aloe vera* gel, antibody titers, immunity, growth performance, broiler

## Abstract

**Objective:**

The experiment evaluated how *Aloe vera* gel (AVG) extract supplementation affected immune responses and physiological performances in broiler chickens.

**Materials and Methods:**

90-day-old Cobb 500 broiler chicks were reared for 38 days without the addition of antibiotics, either through feed or water. At 10 days, chicks were allocated into three groups: A, B, and C (*n* = 30). Group A served as non-supplemented control. Groups B and C were administered aqueous extracts of AVG at 1.0% and 2.0%, respectively, with drinking water.

**Results:**

The supplementation of AVG potentiated the chicken immune response to Newcastle disease-vaccinated birds and sheep red blood cell-treated birds, which detected the highest antibody titers against Newcastle disease virus and sRBC. The cellular immune response evaluated through a cutaneous basophilic hypersensitivity test using phytohemagglutinin-P demonstrated a significant increase in skin thickness in AVG-supplemented birds. The relative sizes of lymphoid organs (bursa, spleen, and thymus) were significantly enhanced (*p* < 0.05) among the groups. Broilers given AVG-1 and AVG-2 exhibited significantly greater (*p* < 0.01) live body weight, dressing percentages, and serum protein and serum albumin levels. The supplemented groups experienced a significant reduction in total serum cholesterol, triglycerides, and low-density lipoprotein–cholesterol values, while the levels of high-density lipoprotein-cholesterol remained unchanged. The dietary aqueous extracts of AVG are effective in enhancing innate and specific immunity.

**Conclusion:**

This work strengthens the perspective of the use of AVG as an immune stimulant to facilitate recovery from immune suppression states, enhance innate and specific immunity, and improve broiler growth performance.

## Introduction

The fast expansion of the poultry industry has increased the possibility of disease outbreaks in commercial poultry flocks. Under these conditions, antibiotics are commonly employed in poultry feeds for therapy and growth enhancement. Prolonged utilization of antibiotics in animal feed is thought to cause a variety of problems, including increasing antibiotic resistance in pathogens, antibiotic residue accumulation in livestock products and surroundings, and a disturbance of normal micro-organisms present [1]. As a result, antibiotic growth promoters in broiler diets are forbidden. Phytogenic feed additives have become more popular as a natural additive in broiler feeds in recent years and have been shown to have growth-promoting, immune-enhancing, and/or therapeutic qualities [2].

*Aloe vera* (AV), scientifically known as *Aloe barbadensis* Miller, is a widely recognized medicinal herb that is utilized for both commercial and therapeutic purposes. It is a member of the Liliaceae family and resembles a cactus in appearance. The leaf is the most important part of the AV, which is made of latex and gel. The gel consists of approximately 98.5%–99.5% water, while the remaining dry matter contains over 75 biologically active components that possess medicinal properties for the treatment of various diseases [3]. Gels containing anthraquinones, saccharides, and other compounds work as antibacterial, antioxidant, and immune modulators [4]. AV as a feed additive to broiler diets improves immune response and growth performance [5]. 1% AV leaf powder increases body weight and can serve as a viable alternative to antimicrobial growth promoters [6]. AV gel contains acemannan, which has the potential to influence humoral and cellular immunity [7].

It stimulates the development of immature dendritic cells, as evidenced by an increase in allogeneic mixed lymphocyte reaction and IL-12 production. AV also inhibited mast cell granulation and enhanced the synthesis of the anti-inflammatory cytokine IL-10 [8]. AV showed positive effects on the humoral immunity of broilers [9] and significantly increased the production of CD4+ and CD8 + T cells and IgG in the rabbit’s blood when injected with the AV vaccine [10]. The ingestion of AV had a positive effect on the composition of lymphocyte subsets and serum immunoglobulins in rabbits [11]. In rats, the aqueous extract of AV markedly boosted the secondary humoral immune response [12]. In a mouse model study, it was found that oral therapy with AV extract at a dose of 100 mg/kg greatly reduced the pyrogallol-induced inhibition of both humoral and cell-mediated immune responses [13].

A neutraceutical combination of AV, Poria cocos, and rosemary extract triggered the activation markers on various types of innate immune cells, resulting in enhanced immune surveillance and antioxidant defense [14]. In C57BL/6 mice, processed AV gel given orally functions as an adjuvant for influenza vaccinations [15–17]. Alprogen and *Aloe* polysaccharides play a role in modulating the immune system and specifically have antiallergic effects [18]. AV could be used for broilers in the form of gel, powder, ethanolic extract, or aqueous extract. Most of the literature found is based on gel, powder, or ethanol extracts of AV. There is limited information available on the effect of an aqueous extract of *Aloe vera* gel (AVG) on immune response in broiler chickens. The research work evaluated the impact of aqueous extracts of AVG on humoral and cellular immune responses in an antibiotic-free broiler, along with their effects on physiological and biochemical parameters.

## Materials and Methods

### Statement of the experiment

The experiment took place in the shed at the Department of Physiology, Bangladesh Agricultural University, Mymensingh. The duration of the experiment spanned from February 25, 2021, to April 5, 2021. 90 Cobb 500 day-old broiler chicks were considered for the study. The research methodology was conducted in compliance with the guidelines of the Animal Welfare and Experimentation Ethics Committee (AWEEC) of Bangladesh Agricultural University, Mymensingh, Bangladesh [AWEEC/BAU/2020-17].

### Experimental protocol and management

The experiment was carried out using a fully randomized design. At the age of 10 days, the chicks were randomly allocated into three groups, namely group A, group B, and group C, each containing 30 birds. The birds in groups A, B, and C were reared separately. Group A was designated as the control group and was provided with regular drinking water and commercial feed without any additional supplements. Group B was administered a 1% concentration of AVG through their drinking water, referred to as AVG-1. Group C was administered a 2% concentration of AVG through their drinking water, referred to as AVG-2. Broiler feeds were purchased commercially. Feed and water were provided *ad libitum,* and feed waste was avoided. Broiler farming techniques were strictly followed, and birds were immunized against infectious diseases. Biosecurity controls were in place during testing.

### Collection and processing of AVG extracts

The extraction of fresh AV leaves was conducted through a water-based cleaning method and manual extraction techniques. The latex was extracted, and subsequently, the gel was collected. Two concentrated infusions were prepared, one with a concentration of 10% and the other with a concentration of 20%. The gel was subjected to homogenization using hot distilled water and subsequently kept at room temperature for a duration of 6–8 h before its utilization.

### Weight measurement and dressing percentage

Bird weights were taken on various days (10th, 17th, 24th, 32nd, and 38th days), and daily feed and water supply were recorded. The weight of the carcass was measured after the feathers were removed, and the dressing percentage was calculated by dividing the carcass weight by the weight of the live body. Organ weights were determined by calculating the proportion of each organ’s weight to the total weight of the live body.

### Blood collection and serum preparation

Sterile tubes were used to collect blood samples from all groups without the addition of an anticoagulant. For serum separation, blood was left to coagulate at room temperature for a period of 2–3 h and was refrigerated at 4°C overnight. Sera were separated and stored for biochemical testing [19].

### Evaluation of humoral immune response

On the 22nd day, each treatment group received intraperitoneal injections of 1 ml of a 10% solution of sheep red blood cells (sRBC) in phosphate-buffered saline (PBS), with five chickens per group. In addition, three chickens per treatment group received an injection of 1 ml of PBS. 2 ml of blood was obtained from the wing vein before the injection, as well as at 4, 8, and 16 days following the injection of sRBC. Blood samples were subjected to centrifugation to separate sera, stored at −20°C, and heat-treated to render the serum complement inactive for 30 min. The hemagglutination assay was conducted by established protocols [20]. The hemagglutination inhibition (HI) test was used to quantify antibody titers against the ND vaccine.

### Determination of cellular immune response

The evaluation of cell-mediated immunity was conducted by measuring the cutaneous basophil hypersensitivity (CBH) reaction to phytohemagglutinin (PHA), a lectin derived from *Phaseolus vulgaris* (PHA-P). On day 32, six healthy broilers per treatment received an intradermal injection of 100 μg of PHA-P in 0.10 ml of sterile PBS, specifically between the third and fourth digits of the right foot. 0.10 ml of PBS solution was injected into the left foot of each bird as a control. The skin’s thickness was assessed using a vernier caliper immediately before the injection, as well as at 12, 24, and 72 h after the injection. The disparity in thickness between the right and left toe webs was utilized to quantify the CBH response [20].

### Serum biochemical studies

Total serum cholesterol, triglycerides, high-density lipoprotein-cholesterol, low-density lipoprotein–cholesterol, albumin, and glucose were measured using a UV spectrophotometer, T80, manufactured by PG Instruments in the United Kingdom [21].

### Statistical analysis

Statistical analysis was performed on all data using a one-way Analysis of Variance (ANOVA) and post-hoc Tukey’s test. The statistical analysis was carried out utilizing the software Graph Pad Prism 8.

## Results and Discussion

### Effects of AVG on the humoral immune response

Table 1 presents the impact of AVG on the antibody titers related to the humoral immune response, as measured by sheep RBC-based assessments. No detectable antibodies against sRBC were observed in the blood samples obtained before the injection of the sRBC solution or in the blood of birds that were injected with PBS. Nevertheless, 4 days following the injection of sRBC, the levels of antibody titers (log2) against sRBC were found to be significantly higher in the treated groups B and C compared to group A. The AVG treatment group exhibited elevated antibody titers against sRBC at both 8 and 16 days (day 16) compared to the control group (Table 1). There was no significant difference observed in the antibody titers between AVG-1 and AVG-2. In general, the levels of antibodies specific to sRBC reached their highest point 8 days following the injection of sRBC. sRBC is a common antigen that relies on T-cell activation for antibody production. Th2 cells stimulate B cell activation and proliferation, enhancing humoral immunity and effectiveness. The report shows AVG administration significantly *(p <* 0.05*)* increased antibody titers against Newcastle disease virus (NDV) in broilers, consistent with a previous study [6,9] indicating peak levels against sRBC in broilers at 28 and 38 days of age.

### Effects on antibody titers against the ND vaccine in broiler chickens

**Figure 1.**
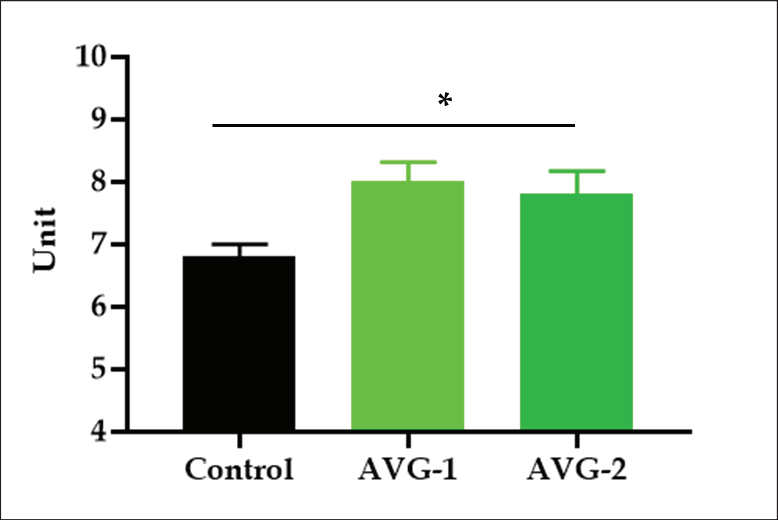
Effects of AVG on antibody titer against ND vaccine in broilers. *Significant at 5% level (control vs. AVG treated group), ns-not significant.

The study used the HI test to measure HI titer values against the ND vaccine in broiler groups. Results showed significantly (*p* < 0.01) higher antibody titers in AVG-1 and AVG-2 broilers (Fig. 1). Daily administration of AVG at two different concentrations to birds vaccinated against ND led to an enhancement of the chicken’s immune response, demonstrating its immune-stimulatory properties. The study confirms previous research [6] showing a significant improvement in antibody titer against NDV in broilers treated with AVG. AV application before infection enhanced humoral responses, and diets supplemented with black cumin seeds and antibiotics showed higher levels of HI titers against NDV [25].

**Table 1.** Effects of AVG on antibody titers against sRBC.

Group	Antibody titer (unit)		
	4th day	8th day	16th day
Control	3.00 ± 0.31^a^	3.43 ± 0.24^a^	3.20 ± 0.37^a^
AVG-1	6.00 ± 0.34^b^	6.80 ± 0.58^b^	7.60 ± 0.50^b^
AVG-2	5.80 ± 0.50^b^	7.00 ± 0.74^b^	7.00 ± 0.63^b^

Values expressed as mean and standard error mean (SEM).

^a,b^different superscript letters in a column denote significant differences at *p* < 0.05 level.

**Table 2.** CBH response (increase in toe web thickness in mm) in broiler chickens fed AVG.

	**Before injection**		**12 h**		**24 h**		**48 h**	
	**PBS**	**PHA-P**	**PBS**	**PHA-P**	**PBS**	**PHA-P**	**PBS**	**PHA-P**
Control	2.14 ± 0.07	2.38 ± 0.04	2.28 ± 0.05	2.85 ± 0.13^a^	2.18 ± 0.07	2.92 ± 0.08^ a^	2.27 ± 0.04	3.56 ± 0.17^ a^
AVG-1	2.05 ± 0.04	2.30 ± 0.06	2.18 ± 0.09	3.80 ± 0.04^b^	2.24 ± 0.36	5.16 ± 0.03^ b^	2.58 ± 0.26	5.45 ± 0.22^ b^
AVG-2	2.24 ± 0.04	2.34 ± 0.01	2.34 ± 0.02	4.79 ± 0.03^c^	2.40 ± 0.63	5.91 ± 0.10^c^	2.40 ± 0.33	5.77 ± 0.46^ b^

Values as mean and SEM. ^a,b,c^superscript letters in a column differ significantly (*p* < 0.05).

### Cellular immunity in broiler chickens by the CBH test

The cellular immunity of broiler chickens fed AVG was assessed by CBH response (Table 2). The CBH response was triggered by the PHA-P before, 12, 24, and 48 h post-injection. The study demonstrated that there was a time-dependent increase in skin thickness in the right toe web area treated with PHA-P compared to the left foot treated with PBS (*p* < 0.05). The administration of AVG resulted in varying levels of CBH responses. The data provided substantiated that the administration of AVG resulted in heightened immune responses in avian species. The current results align with the findings reported in a previous study [24], which demonstrated that daily 1% AVG boosts both cellular and humoral immunity in chickens and increases chemokinetic activity and stronger response of their splenic lymphocytes to the mitogen PHA in mice. In addition, there was an enhancement of anti-SRBC antibody production [25]. Females show a higher T cell blastogenic response compared to males, possibly due to multiple gene differences in response [26]. In contrast to the humoral immune response, previous research has indicated a positive correlation between body weight and the response to PHA-P [27].

### Relative lymphoid organs and live body weight

**Figure 2.**
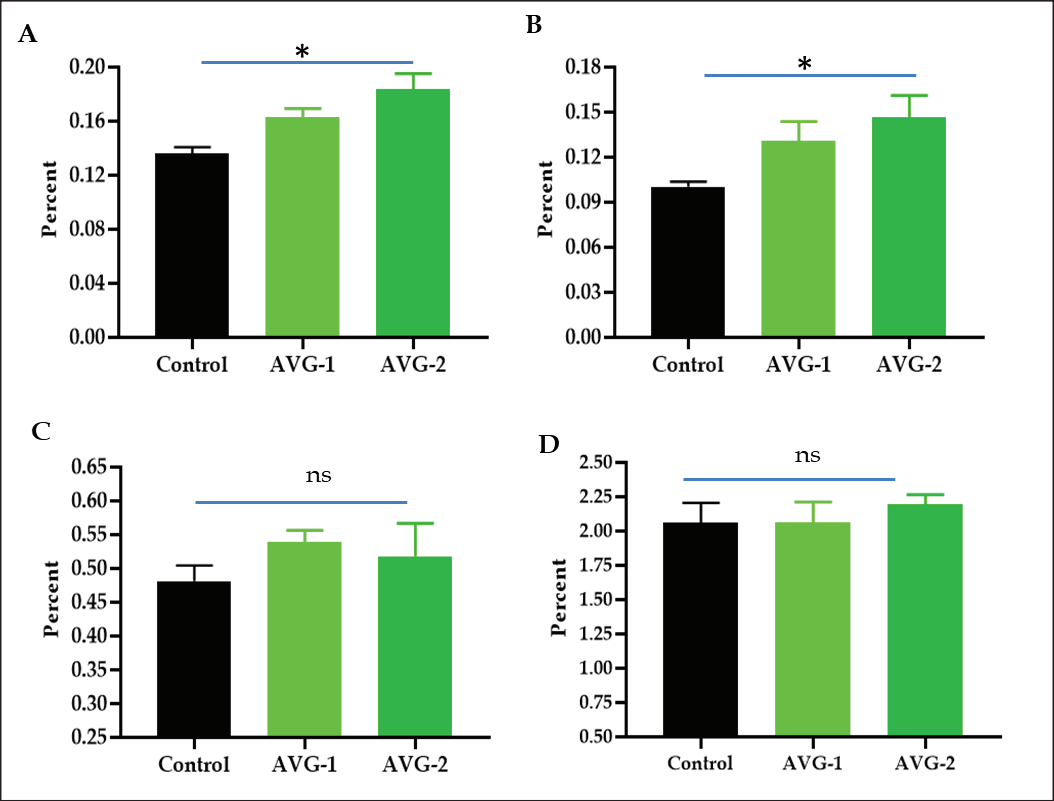
Lymphoid organ weight (%) relative to body weight of broiler chickens fed two different concentrations of *A. vera* gel extracts. (A), spleen, (B) bursa of Fabricius, (C) thymus, and (D) liver. *Significant at 5% level (control *vs.* AVG treated group), ns-not significant.

**Figure 3.**
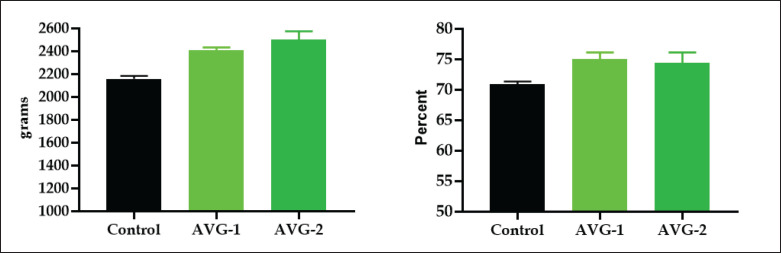
Effects of AVG on live body weight gain (gm) and dressing percentage in broilers (Mean ± SEM) in broiler chickens. (A) Live body weight gain. (B) Dressing percentage. *Significant at *p* < 0.05.

Figure 2 shows increased relative weights of lymphoid organs (spleen, bursa, and thymus) and non-lymphoid organs (liver) in AVG-1 and AVG-2-treated groups compared to the control group. The study confirms previous research showing increased spleen and bursa mass in broilers [8], increased splenic lymphocytes in mice [25], and the presence of acemannan in *Aloe* gel, which activates macrophages and T cells [27]. Birds fed diets containing herbs and antibiotics showed a significant elevation (*p* < 0.05) in the ratio of lymphoid organ weight to body weight [24]. The current experiment showed a gradual increase in bird body weights across all groups, with a significant increase in the final 3 weeks in the AVG-treated groups (Fig. 3). On the 38th day, the experiment showed significant variation (*p* < 0.05) in live body weight between group A (2158.50 ± 23.8 gm) and treatment groups B (2412.50 ± 19.2 gm) and C (2502.7 ± 33.4 gm). The body weights were recorded as highest in groups B (1%) and C (2%). The observed accelerated body weight gain in groups B and C can be attributed to improved utilization of feed and provided nutrients. This effect is likely owing to the inclusion of a growth promoter and AV gel in the diet, which plays a crucial role in promoting overall health and facilitating body weight gain. The previous findings [8] showed that the AVG groups exhibited greater increases in body weight and feed intake. Birds given drinking water containing AVG showed significant improvements in final body weight, weight gain, daily weight gain, and feed conversion ratio (*p* ≤ 0.05) [28]. The study found no significant difference between the 1% and 2% AVG treatment cohorts, but dressing percentages increased in groups B and C, consistent with previous findings [29]. Drinking water supplemented with herbal growth promoters improved feed utilization in broiler chickens, resulting in significant body weight gain and a higher profit margin. 1% and 2% AVG supplementation yielded higher profitability.

### Effects of AVG on biochemical parameters in broilers

The study found significant differences (*p* < 0.05) in total cholesterol levels between group A (190.62 ± 6.59 mg/dl) and treatment groups B (161.14 ± 7.81 mg/dl) and C177.45 ± 13.58 mg/dl, with AVG significantly affecting serum cholesterol levels (Table 3). Triglyceride levels also varied between groups, with B having higher values and C having lower values. The study found no significant difference in HDL cholesterol levels between the control and treatment groups. The LDL cholesterol levels varied (*p* ≤ 0.05) among the three groups: control (127.62 ± 3.38 mg/dl), treatment group B (94.33 ± 2.89 mg/dl), and group C (112.27 ± 1.75 mg/dl) (Table 3). The consumption of 1.5% AVG in drinking water led to a significant decrease in total blood cholesterol, triglycerides, and LDL levels among the birds in the experimental group [29]. The study found higher HDL levels in birds, contradicting previous studies that found no notable disparities in total cholesterol, triglycerides, HDL, and LDL levels between treatment and control groups when AV and turmeric were administered [30].

Broiler birds receiving 1% and 2% AVG showed non-significantly higher levels of total protein, albumin, and glucose compared to the control group (Table 4). The current findings align with the earlier findings [29,31]. The addition of AVG to drinking water resulted in enhanced biochemical parameters in broilers. Blood biochemical parameters are crucial biomarkers for health status and metabolic processes, indicating immune status, metabolic rate, and fat metabolism. The levels of these derivatives must remain within the normal range in birds for good health. However, these results contradict the findings of [31,32], who reported no significant variations in birds fed a diet containing AV. The inconsistent findings can likely be attributed to variations in the administration of AV incorporated into the drinking water in the current investigation. Broilers fed AVG through drinking water showed reduced triglycerides, total cholesterol, and LDL levels, consistent with previous reports [29,33]. Acemannan, a key polysaccharide in AVG, plays a crucial role in regulating blood cholesterol levels through lipid metabolism regulation. The study’s weak points lie in the need to investigate the specific components of AVG that influence immune responses and the expression levels of various subsets of B cells and T cells.

**Table 3.** Effects of AVG on lipid profile in broilers.

Groups	Total cholesterol (mg/dl)	Triglyceride (mg/dl)	HDL (mg/dl)	LDL (mg/dl)
Group A (Control)	190.62 ± 6.59^a^	121.85 ± 16.52^a^	38.63 ± 0.56^a^	127.62 ± 3.38^a^
Group B (AVG-1)	161.14 ± 7.81^b^	91.98 ± 7.96^b^	39.79 ± 1.17^a^	94.33 ± 2.89^b^
Group C (AVG-2)	177.45 ± 13.58^b^	97.67 ± 8.45^b^	40.48 ± 3.53^a^	112.27 ± 1.75^c^

^a,b,c^ ^superscript letters in a column differ significantly (^*p*^ < 0.05).^

**Table 4.** Effects of AVG on total protein, albumin, and serum glucose in broilers.

Groups	Total protein (gm/dl)	Albumin(gm/dl)	Glucose(mg/dl)
Group A (Control)	3.91 ± 0.25^a^	2.03 ± 0.11^a^	100.45 ± 6.41^a^
Group B (AVG-1)	4.03 ± 0.41^a^	1.96 ± 0.23^ a^	121.59 ± 10.68^a^
Group C (AVG-2)	4.45 ± 0.22^a^	1.88 ± 0.12^a^	137.93 ± 6.45^a^

^a,b,c^ ^superscript letters in a column differ significantly (^ *p* ^< 0.05).^

## Conclusion

The study suggests that aqueous extracts of AVG enhance physiological performance and immune responses in broiler birds, but more detailed molecular studies with known AVG concentrations are needed.

## List of abbreviations

ANOVA, analysis of variance; AVG, *Aloe vera gel*; CBH, cutaneous basophilic hypersensitivity; NDV, Newcastle disease virus; PBS, phosphate- buffered saline; IgG, immunoglobulin G; PHA, phytohemagglutinin A; SEM, standard error mean; sRBC, sheep red blood cells.
